# Comprehensive assessment of actionable genomic alterations in primary colorectal carcinoma using targeted next-generation sequencing

**DOI:** 10.1038/s41416-022-01913-4

**Published:** 2022-07-16

**Authors:** Yi-Hua Jan, Kien Thiam Tan, Shu-Jen Chen, Timothy Tak Chun Yip, Cu tai Lu, Alfred King-yin Lam

**Affiliations:** 1 Cancer Genomics, ACT Genomics, Co. Ltd., Taipei City, Taiwan; 2 Medical Science, ACT Genomics, Co. Ltd., Hong Kong SAR, China; 3 Department of Surgery, Gold Coast University Hospital, Southport, QLD 4215 Australia; 4 Cancer Molecular Pathology, School of Medicine and Dentistry, Menzies Health Institute Queensland, Gold Coast campus, Griffith University, Gold Coast, QLD 4222 Australia; 5 Pathology Queensland, Gold Coast University Hospital, Southport, QLD 4215 Australia

**Keywords:** Cancer genomics, Gastrointestinal cancer

## Abstract

**Background:**

The clinical utility of comprehensive genomic profiling (CGP) for guiding treatment has gradually become the standard-of-care procedure for colorectal carcinoma (CRC). Here, we comprehensively assess emerging targeted therapy biomarkers using CGP in primary CRC.

**Methods:**

A total of 575 primary CRCs were sequenced by ACTOnco® assay for genomic alterations, tumour mutational burden (TMB), and microsatellite instability (MSI).

**Results:**

Eighteen percent of patients were detected as MSI-High (MSI-H), and the remaining cases were classified as microsatellite stable (MSS). Driver mutation prevalence in MSS CRCs were *APC* (74%), *TP53* (67%), *KRAS* (47%), *PIK3CA* (21%) and *BRAF* (13%). The median TMBs for MSI-H and MSS patients were 37.8 mutations per mega base (mut/Mb) and 3.9 mut/Mb, respectively. Forty-seven percent of MSI-H CRC harboured at least one loss-of-function mutations in genes that may hamper immune checkpoint blockade. Among MSS RAS/RAF wild-type CRCs, 59% had at least one actionable mutation that may compromise the efficacy of anti-EGFR therapy. For late-stage CRC, 51% of patients are eligible for standard care actionability and the remaining 49% could be enrolled in clinical trials with investigational drugs.

**Conclusions:**

This study highlights the essential role of CGP for identifying rational targeted therapy options in CRC.

## Background

Colorectal carcinoma (CRC) is one of the leading causes of cancer mortality worldwide [1]. In addition to surgery, radiation therapy and combination chemotherapy (e.g. folinic acid, fluorouracil and oxaliplatin) have been the standard chemotherapy treatment strategy for patients with metastatic CRC in the past decades and resulting in median overall survival ranging from 18 to 20 months [2]. Chemotherapies in combination with vascular endothelial growth factor (VEGF) inhibitors and epidermal growth factor receptor (EGFR) inhibitors further extended the median survival of patients with metastatic CRC to 30 months [2]. Although the survival rate has improved, the progress of developing more therapeutic options for patients with metastatic CRC remained slow until recent advances in genomic biomarkers-guided therapies [3].

With the increasing implementation of next-generation sequencing (NGS) technologies in clinical oncology, the number of actionable genomic alterations in oncology has expanded in recent years [4]. For example, about 70% of *RAS* wild-type CRCs simultaneously harbour heterogeneous genomic alterations involved in epidermal growth factor receptor (EGFR) and other signalling pathways that confer resistance to anti-EGFR monoclonal antibodies (mAb) therapy [5]. In addition, patients with CRCs that are microsatellite instability (MSI) and high somatic tumour mutation burden (TMB) have shown encouraging outcomes after receiving immunotherapy including one of the single-agent programmed death-ligand 1 (PD-L1) checkpoint inhibitors [6, 7]. Aside from MSI-high CRCs, around 60% of patients with CRC could benefit from single or combined targeted therapies [8]. Moreover, microsatellite stable (MS) tumours harboured DNA polymerase-ε (*POLE*) mutation result in ultra-high TMB and neoantigen loads that are responsive to PDL-1 monoclonal antibody (mAb) therapy [9–11].

In this study, we aim to investigate the landscape of genomic alterations that are associated with the standard-of-care treatments and analyse the feasibility of applying potentially actionable genes to fill unmet need for developing potential therapies for patients with CRC.

## Methods

### Specimens and patient consent

All formalin-fixed paraffin-embedded (FFPE) specimens were collected from consecutive patients with primary colorectal adenocarcinoma who underwent resection in hospitals in Queensland, Australia. The cancer samples were recruited with the IRB approval (MED/05/06/HREC) by Griffith University Human Research Ethics Committee. Informed consent was obtained from all subjects. For each cancer sample, the tumour region is selected to ensure the maximum amount of tissue (>70%) for DNA extraction by a pathologist (AKL) accredited by The Royal College of Pathologists of Australasia (RCPA) and The Hong Kong College of Pathologists. The clinical and pathological information (age, sex, location of primary tumour, grade, pathological stage) were confirmed and reviewed by the authors in a multidisciplinary team meeting (CTL and AKL).

### Targeted next-generation sequencing (NGS) workflow

The entire workflow was performed in a College of American Pathologist (CAP)-accredited NGS laboratory at ACT Genomics. Briefly, genomic DNA (gDNA) extracted from the selected FFPE samples of CRC was sequenced to detect single-nucleotide variants (SNV), small insertions and deletions (indels) and copy-number variations (CNV) using ACTOnco^®^ comprehensive cancer gene panel. ACTOnco^®^ encompasses the coding region of 440 cancer-related genes that inform cancer treatment and prognosis of patients with cancer. Forty nanograms of gDNA were amplified with four pools of primer pairs and the library was prepared using the Ion AmpliSeq Library Kit (Thermo Fisher Scientific, Waltham, MA, USA). Amplicons were ligated with barcoded adaptors using the Ion Amplicon Library Kit (Life Technologies, Carlsbad, CA, USA). Barcoded libraries were subsequently conjugated with sequencing beads by emulsion polymerase chain reaction (PCR) and enriched using IonChef (Life Technologies) according to the Ion Torrent protocol (Life Technologies). The quality and quantity of amplified libraries were determined using the AATI fragment analyser (Agilent, Santa Clara, CA, USA) and Qubit (Invitrogen, Waltham, MA, USA). Sequencing was performed on the Ion Proton sequencer using the Ion PI chip (Life Technologies) according to the manufacturer’s protocol.

Sequencing raw reads were mapped to the hg19 reference genome (version 5.10). Single-nucleotide variants (SNVs) and indels (an insertion or deletion mutation involving a sequence of nucleotides) were identified by the Torrent Variant Caller plug-in (version 5.10). Variant Effect Predictor (VEP, version 88) was used to annotate every variant with databases from COSMIC v.86 and Genome Aggregation database r2.0.2. Criteria for further variant analysis were at least 25 variant reads, and an allele frequency of at least 2% for actionable and 5% for other variants. Variants with an allele frequency of at least 1% in Genome Aggregation database r2.0.2 were disregarded as polymorphisms, and technical errors removed with the ACT Genomics in-house sample database of healthy volunteers.

Copy-number variations (CNVs) were processed using the following steps. First, amplicons with read counts in the lowest 5th percentile and those with a coefficient of variation ≥0.3 were removed. To correct the samples generated from different amplicon pool designs, each pool was normalised. Then, ONCOCNV [12] was applied to normalise total amplicon number, GC content of each amplicon region, amplicon length, and technology-related biases, and to segment the sample with a gene-aware model. These methods were applied for establishing the baseline (24 peripheral blood mononuclear cell samples) and on the sample data. Aberration Detection in Tumour Exome (ADTEx) was applied for correcting baseline shifts and estimating tumour purity using the change ratio of all loss of heterozygosity (LOH) and allele-specific copy-number analysis (ASCNA) in pooled single-nucleotide polymorphisms (SNPs) data. Copy-number amplification was defined as copy number ≥4, whereas copy number loss was defined as copy number ≤1. Copy-number loss estimation was not provided for the samples with tumour purity less than 30%.

### Clinical actionability assessment

Clinical actionability was assessed according to OncoKB and ACT Genomics in-house knowledge database. Mutations were classified into four tiers according to their level of evidence. Briefly, all United States Food and Drug Administration (FDA)-recognised biomarkers that are predictive of response to FDA-approved drugs in specified indications are regarded as Level 1. Biomarkers that predict standard-of-care therapies in specified indications are regarded as Level 2. Biomarkers that predict response to therapies approved by the FDA or professional societies in a different indication are classified as Level 3A, whereas prospective or retrospective biomarkers for clinical trials are Level 3B. Biomarkers that show plausible therapeutic benefits based on clinical studies, case reports, or pre-clinical studies are classified as Level 4. Levels 1 and 2 are considered on-label indications, whereas Levels 3 and 4 are regarded as off-label indications.

### Microsatellite instability determined by targeted NGS

Sequence diversity of more than 500 genomic loci were selected to identify the status of the microsatellite instability of the given sample. To estimate the variation of sequence features of clinical samples, a set of normal samples were included to build the baseline features of selected loci. To minimise the bias derived from sequencing error, we eliminated the loci with high variability across repeat sequences. About 400 genomic loci across the sequencing regions are then used to estimate the microsatellite instability levels of clinical samples. Genomic patterns of the selected loci of pan-cancer samples of microsatellite stable cohort and of microsatellite instability cohort were applied to train the in-house microsatellite model by machine learning. The microsatellite status (stable or instability/high) of clinical samples will be identified by the in-house model with the genomic features of the selected loci.

## Results

### Cohort characteristics

To inform potential targeted therapy options, we sequenced a total of 575 primary CRCs using ACTOnco® assay that was designed to detect SNV, small INDELS and CNV in 400+ cancer-related genes. Study cohort characteristics including age, gender, pathological stage, grade, and location of the primary tumour, and are summarised in Table 1. Briefly, the median age of the cohort was 67 years and 46.3% were female. Of the 575 CRCs, 53.2% were early Stage (I or II) and 46.8% were late Stage (III or IV). As for the location of the primary tumour, 45.6% were derived from the right side of the colon (caecum, ascending colon, transverse colon) and the remaining 54.4% were left side (descending colon, sigmoid colon or rectum) lesions. Well-, Moderately- and poorly differentiated tumours account for 26.3%, 62.8% and 10.9% of the entire cohort, respectively.

**Table 1:** Clinicopathological characteristics of patients with colorectal carcinoma included in the analysis.

Characteristics	All subjects 575 (100%)
Age	
Years (mean ± standard deviation)	67.0 ± 12.7
Sex	
Male	309 (53.7%)
Female	266 (46.3%)
Stage	
I	125 (21.7%)
II	181 (31.5%)
III	159 (27.7%)
IV	110 (19.1%)
Primary tumour location	
Right colon	262 (45.6%)
Left colon	199 (34.6%)
Rectum	114 (19.8%)
Primary tumour grade	
Well differentiated (grade 1)	151 (26.3%)
Moderately differentiated (grade 2)	361 (62.8%)
Poorly differentiated (grade 3)	63 (10.9%)

### Genomic alteration landscape

**Fig. 1:**
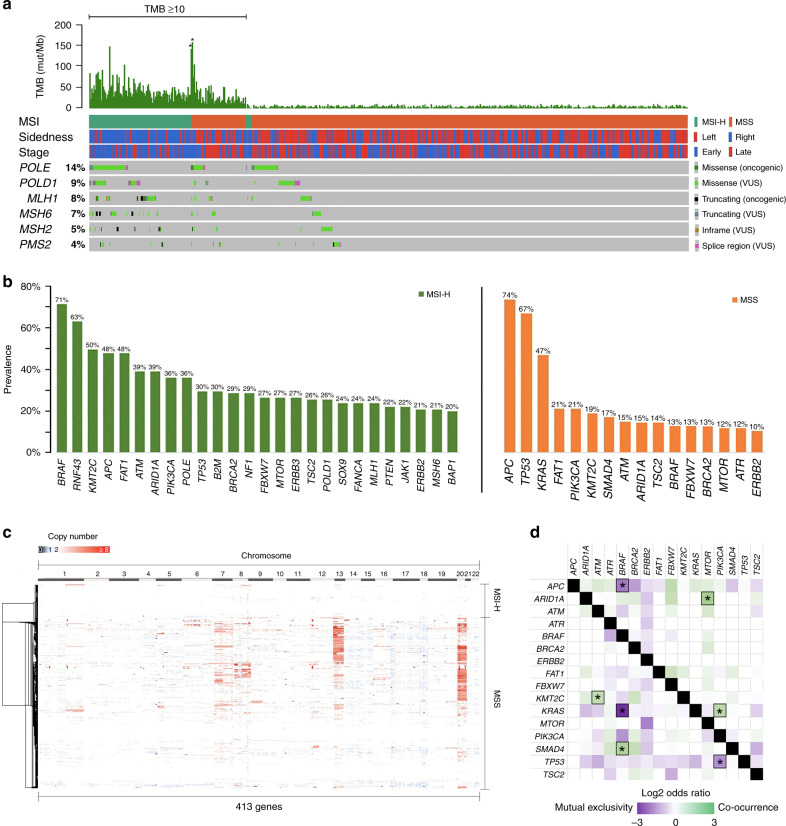
Landscape of genomic alterations in 575 primary CRCs. **a** Mutations in *POLE*, *POLD1*, mismatch-repair genes in NGS-based MSI subtyping of primary CRCs. *POLE oncogenic driver mutation. **b** Prevalence of frequently mutated oncogenes/tumour suppressor genes in MSI-H and MSS CRCs. **c** Copy-number (CN) landscape of MSI-H and MSS CRCs. Genes affected by CN gain or deep loss were colour-coded with red (CN >= 4) or blue (CN < 1) gradient, respectively. **d** Co-occurrence and mutual exclusivity analysis of recurrent oncogenes and tumour suppressor genes in MSS CRCs. *P < 0.01.

A total of 17,810 variants in 428 genes were identified, including 13,685 missense, 2549 truncating, 1293 splice region, and 285 in-frame variants. To classify CRC subtypes and their corresponding tumour mutational burden (TMB), we utilised NGS-derived algorithms to estimate the TMB and stratify patients into MSI-H or MSS subtypes (Fig. 1a). We also evaluated mutations in genes encode DNA polymerase catalytic subunits (*POLE* and *POLD1*) and DNA mismatch-repair genes (*MLH1*, *MSH6*, *MSH2*, and *PMS2*). In the MSI-high (MSI-H) CRCs, *BRAF* (71%) was the most frequently mutated oncogene followed by *RNF43 (63%)*, *KMT2C (50%)*, *APC (48%)*, *FAT1 (48%)*, *ATM* (39%) and *ARID1A (39%)* tumour suppressor genes (Fig. 1b). On the other hand, in MSI stable (MSS) CRCs, *APC* (74%) and *TP53* (67%) were the most frequently mutated tumour suppressor genes and *KRAS* (47%), *PIK3CA* (21%) and *BRAF* (13%) were among the most mutated oncogenes (Fig. 1b). Copy-number landscape of 413 genes revealed that genes located at chromosome 7p and q, 8p and q, 13q and 20q including *EGFR* (07p11.2, 12.3%)*, MYC* (08q24.21, 12.1%), *FLT1* (13q12.3, 30.6%)*, FLT3* (13q12.2, 31.0%)*, SRC* (20q11.23, 37.9%)*, AURKA* (20q13.2, 36.8%)*, GNAS* (20q13.32, 36.8%)*, ZNF217* (20q13.2, 43.8%) and *BCL2L1* (20q11.21, 50.6%) were frequently amplified in MSS tumours (Fig. 1c). Co-occurrence and mutual exclusivity analysis of MSS CRC samples identified four significant (*P* < 0.01) co-occurrent gene pairs (*KRAS/PIK3CA, ARID1A/MTOR, KMT2C/ATM* and *SMAD4/BRAF*) and three significant (*P* < 0.01) mutual exclusive gene pairs (*APC/BRAF, KRAS/BRAF* and *TP53/PIK3CA*) in the recurrent mutated oncogenes and tumour suppressor genes (Fig. 1d).

### NGS-derived classification of MSI and TMB

**Fig. 2:**
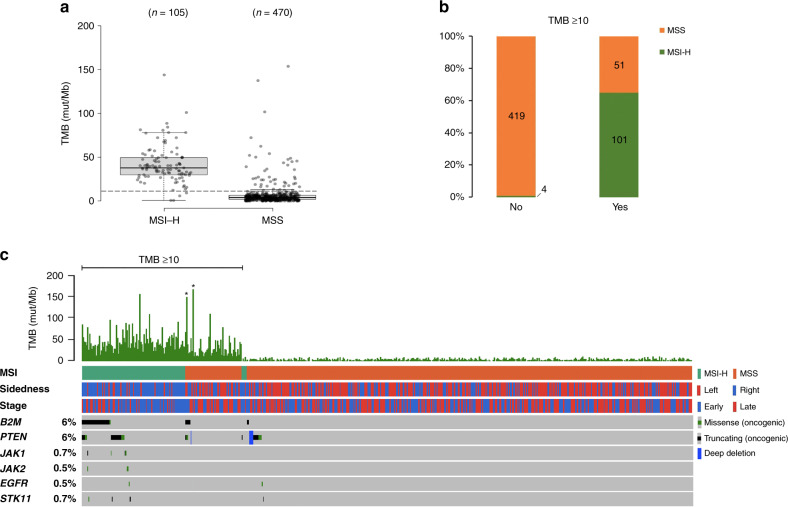
Putative ICI-resistant mutations in MSI-H/TMB-H and MSS/TMB-H CRCs. **a** Comparison of TMB distribution between MSI-H and MSS CRCs. **b** Precent comparison of MSI-H and MSS composition between tumours with TMB≥10 and those with TMB < 10. **c** Mutations in putative ICI-resistant biomarkers in MSI-H and MSS CRCs.

We next analysed and compared the tumour mutational burden (TMB) distributions in MSI-H and MSS CRCs. The median TMBs for MSI-H and MSS patients were 37.8 mutations/Mb and 3.9 mutations/Mb, respectively (Fig. 2a). Among CRCs derived from the left side of the colon, only 5.6% were MSI-high tumours. In contrast, one-third of right-sided tumours were MSI-high. In MSS group, we identified 51 CRCs with TMB ≥ 10.0 mutations/Mb, which account for 8.9% of the entire study cohort including 2 *POLE* mutant tumours (Fig. 2b). TMB-high (≥10 mutations/Mb) has been suggested as a pan-cancer criterion for selecting patients who may be responsive to immunotherapy. In this cohort, 152 (26.4%) samples were identified as TMB-high, and 51 (34.9%) patients of this subgroup were classified as MSS (Fig. 2b). To identify the percentage of genomic defects in components of antigen presentation machinery and immune response regulation in MSI-H CRCs, we analysed series of truncating or oncogenic mutations in *B2M, PTEN, JAK1, JAK2, STK11* and *EGFR*, that were previously reported to be associated with immune checkpoint inhibitor resistance and identified 46.7% of TMB-high CRC harboured at least one oncogenic mutation in those genes that may lead to immune checkpoint inhibitor resistance (Fig. 2d).

### Spectrum of actionable alterations and pathways by CRC subtypes

**Fig. 3:**
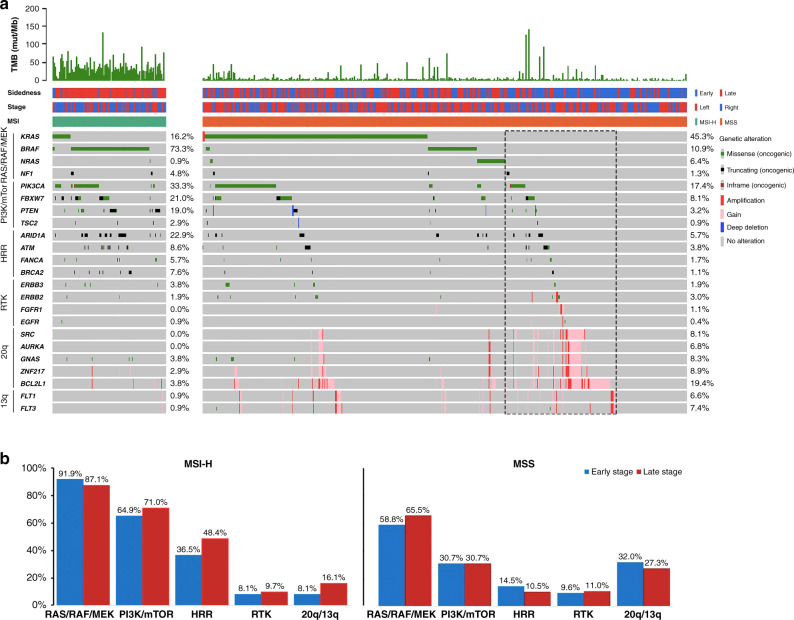
Landscape of actionable oncogenic alterations in MSI-H and MSS CRCs. **a** Actionable mutations grouped by RAS/RAF/MEK, PI3K/mTOR, HRR and RTK pathways. Percentage of patients with wild-type *RAS/RAF* plus at least one off-label indication was framed with dash line. **b** Comparison of percent pathway altered between the early-stage and the late-stage CRCs in MSI-H and MSS subtypes.

To analyse the composition of targetable pathways, we compared the prevalence of actionable alterations by signalling pathways between MSI-H and MSS CRCs. Regardless of the clinical stages, 94% of MSI-H CRCs harboured at least one potentially actionable alteration compared to 63% of MSS CRCs (Fig. 3a; *P* < 0.01). The MSI-H-enriched oncogenic mutations include *BRAF* (73.3% versus 10.9%, *P* < 0.01), *PIK3CA* (33.3% versus 17.4%, *P* < 0.01), *FBXW7* (21.0% versus 8.0%, *P* < 0.01), *PTEN* (19.0% versus 3.2%, *P* < 0.01), *ARID1A* (22.9% versus 5.7%, *P* < 0.01), and *BRCA2* (7.6% versus 1.1%, *P* < 0.01) (Fig. 3a). On the other hand, *KRAS* and *NRAS* oncogenic mutations were significantly enriched in MSS CRCs compared with MSI-H CRCs (45.3% versus 16.2%, *P* < 0.01; 6.4% versus 0.9%, *P* < 0.01) (Fig. 3a). Moreover, it is worth noted that gene copy-number gain/amplification in chromosome 20q (*SRC*, *AURKA*, *GNAS*, *ZNF217*, and *BCL2L1)* and 13q (*FLT1* and *FLT3*) were enriched in CRCs with *RAS/RAF* wild-type MSS CRCs compared with CRCs harboured oncogenic *RAS/RAF*. Furthermore, *MYC* amplification was found to be significantly associated with the late-stages MSS CRCs compared with the early-stages MSS CRCs (Table 2). As a result, 59% of *RAS/RAF* wild-type MSS CRCs harboured at least one actionable alteration in *NF1, 20q, 13q*, PI3K/mTOR, receptor tyrosine kinase (RTK) or homologous recombination repair (HRR) pathways that may compromise anti-EGFR therapy (Fig. 3a). With respect to the actionable pathways, PI3K/mTOR and HRR pathways were significantly altered in MSI-H CRCs compared with MSS CRCs, and the percentage of altered HRR pathways were significantly higher in the late-stages MSI-H CRCs compared to those in the early stages (Fig. 3b).

**Table 2:** Comparison of Copy-number variation (CNV) characteristics of patients with microsatellite stable (MSS) cancers in early- and late stage.

Cytoband/gene	Early stage, *n* = 233	Late stage, *n* = 239	*P* value
08q22.3/UBR5 gain			0.0575
Yes	14 (6.0%)	26 (10.9%)	
No	219 (94.0%)	187 (89.1%)	
08q24.21/MYC gain			0.0107
Yes	17 (7.3%)	35 (14.6%)	
No	216 (92.7%)	204 (85.4%)	
08q22.1/CCNE2 gain			0.0700
Yes	16 (6.9%)	28 (11.7%)	
No	217 (93.1%)	211 (88.3%)	

### Assessment of clinical actionability and drug combination in recurrent late-stage CRCs

**Fig. 4:**
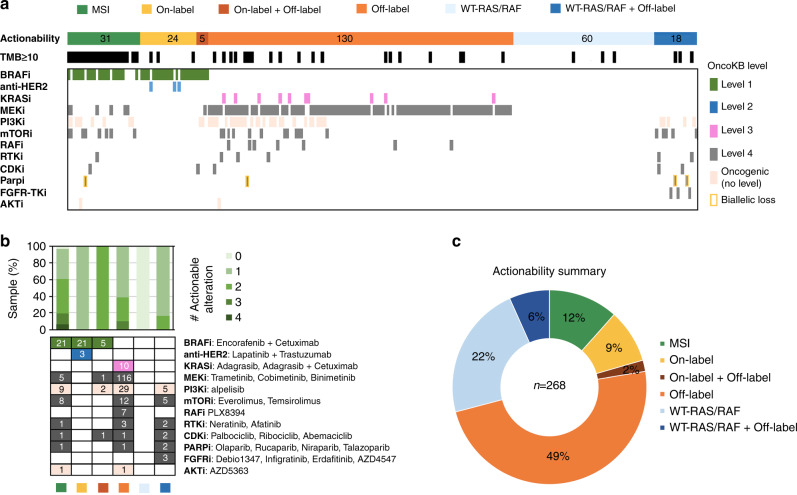
Comprehensive assessment of clinical actionability of recurrent late-stage CRCs. **a** Landscape of biomarker-guided drug grouping in late-stage CRCs. The actionable mutations are grouped by their corresponding targeted therapy drug type and broken down by OncoKB level of evidence in CRC. Samples are presented according to their highest level of evidence of actionability. **b** Upper portion, percentage of samples with a given number of actionable genomic alterations per CRC actionability category. Lower portion, frequencies of actionable alterations in biomarker-guided drug grouping per CRC actionability category. **c** Percentage summary of actionable categories matched to the data from the OncoKB database.

**Fig. 5:**
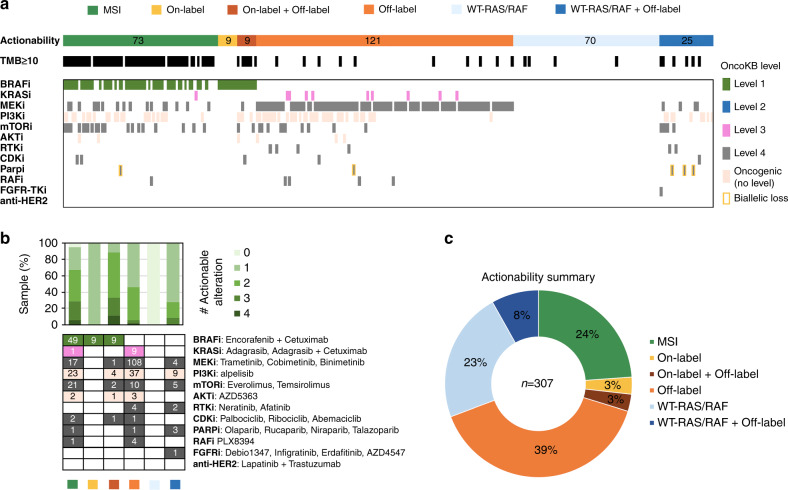
Comprehensive assessment of clinical actionability of genomic markers for colorectal carcinoma (CRC) of early stages. **a** Landscape of biomarker-guided drug grouping in early-stage CRCs. The actionable mutations are grouped by their corresponding targeted therapy drug type and broken down by OncoKB level of evidence in CRC. Samples are presented according to their highest level of evidence of actionability. **b** Upper portion, percentage of samples with given number of actionable genomic alterations per CRC actionability category. Lower portion, frequencies of actionable alterations in biomarker-guided drug grouping per CRC actionability category. **c** Percentage summary of actionable categories matched to the data from the OncoKB database.

To comprehensively assess every actionable alteration in patients with recurrent late-stage (Stage III or IV) CRCs, we first stratified patients into six categories namely MSI, On-label, On-label plus Off-label, Off-label, WT-*RAS/RAF*, and WT-*RAS/RAF* plus Off-label in accordance with their biomarkers. Patients whose tumours with TMB  ≥10.0 were also indicated. We then grouped actionable alterations by drug type and sorted according to the OncoKB levels of evidence (Fig. 4a). Among 29 patients with on-label biomarkers (*BRAF*^V600E^ and *ERBB2* amplification), five of them (17.2%) were found to harbour concurrent actionable alterations that provide alterative off-label therapies including PI3Ki, mTORi, MEKi and CDKi (Fig. 5a). Moreover, for 78 late-stage CRCs with wild-type *RAS/RAF*, eighteen were identified with additional oncogenic mutations that could bypass anti-EGFR blockade (Fig. 4a). For the patients with HRR gene alterations, only those with biallelic loss of HRR genes were considered eligible for PARP inhibitor treatment (Fig. 4a). Beyond MSI CRCs, 63% of patients had at least two potentially actionable alterations (Fig. 4b). For MSI-H CRCs, over 90% had at least one actionable alteration other than immunotherapy (Fig. 4b). In summary, 51% of patients are eligible to standard care actionability (MSI, WT-*RAS/RAF* and on-label biomarkers) and 49% of patients could be enrolled in clinical trials with investigational therapies (Fig. 4c).

### Assessment of clinical actionability and drug combination in early-stage CRCs

Currently, surgical resection followed by chemotherapies remains the standard practice for treating early-stage CRCs, and the benefit of molecularly targeted therapy and immune checkpoint blockade is still uncertain. However, for the early-stage CRC patients with unfavourable clinical and pathological parameters, the identification of actionable biomarkers for screening those who might benefit from targeted therapy in adjuvant settings is still necessary. Therefore, we also performed a comprehensive assessment of clinical actionability for the patients with Stage I or II CRCs and found a similar actionability landscape in comparison to patients with late-stage CRCs (Fig. 5).

## Discussion

In the past decade, large-scale sequencing studies have defined the genetic basis, consensus molecular subtypes, and key signalling pathways of CRC [3, 13]. These findings also reveal the genomic complexity and heterogenous nature of CRC. Here, we prospectively sequenced 575 primary CRCs by using a large panel of targeted sequencing assay to comprehensively assess mutations, copy-number alterations, TMB and MSI status. Collectively, we aim to comprehensively prioritise targeted therapy options with an emphasis on coexisting actionable alterations for patients with CRC.

Current guideline for anti-EGFR therapy relies on negative predictive biomarkers that select patients without activating mutations in *KRAS* and *NRAS*. Efforts have been made to identify resistance mechanisms to anti-EGFR therapy such as *BRAF* mutation, *HER2* amplification, and c-*MET* amplification by means of NGS in metastatic CRC [14, 15]. In addition, genomic alterations act in concert with the EGFR pathway such as *PIK3CA* exon 20 mutations and *PTEN* alterations, also seem to associate with primary resistance to anti-EGFR mAbs therapies [16, 17]. Using comprehensive genomic profiling, we identified 59% of *RAS/RAF* wild-type MSS CRC concurrently harboured at least one oncogenic alteration in *NF1*, 13/20q, PI3K/mTOR, HRR, RTK pathways (Fig. 3a). Our data also showed that tumours deriving from the left side of the colon or rectum had significantly higher mutation prevalence in *APC* and *TP53* and lower mutational burden compared to right-sided tumours. On the other hands, over 80% of tumours originating from the right side of the colon, harboured actionable mutations of *RAS* or *PI3K* pathway compared with less than 60% of cases with left-sided tumours. These data are consistent with other large-scale sequencing studies showing that right-side primary CRCs are often associated with MSI-H and *BRAF*^V600E^ mutation and the oncogenesis of the right-side CRC may rely on RAS and PI3K pathways rather than native receptor tyrosine kinase signalling [18, 19].

MSI-H has been proven as a promising biomarker that predicts benefit from immune checkpoint inhibitor (ICI) in advanced CRC and other types of solid tumours [20, 21]. However, ICI response rates varied from 30 to 50% suggesting the existence of intrinsic resistance factors that ultimately lead to immune evasion. Although inconclusive, numbers of ICI-resistant markers have been proposed for MSI-H tumours, including low TMB, Janus kinase (*JAK1/2/3*) mutations, loss of beta-2-microglobulin (*B2M*) that could impair antigen presentation by class I major histocompatibility complex [6, 20, 22, 23]. In this cohort, 29% of patients with MSI-H tumours harboured truncating mutations in *B2M* and about *7%* of them have at least one mutation in *JAK1* and *JAK2*. In addition, we identified 18.3% of MSI-H tumours carried loss-of-function mutations in *PTEN* that have been linked to the immunosuppressed tumour microenvironment [24]. These data point out the possibility of performing large panel targeted sequencing to optimise patient selection in MSI-H CRC prior to ICI treatment. Similar to MSI-H CRCs, *POLE/POLD1*-altered MSS CRCs showed favourable patients’ prognosis in response to immune checkpoint blockade [25]. Concurrent assessment of MSI and TMB to classify patients into MSI-H/TMB-H and MSS/TMB-H subgroups are essential as the later may expand the population of CRC who may benefit from immune checkpoint inhibitor. However, the definition of TMB-H in MSS CRC remains to be determined. A large cohort study reported cut-off for TMB-H (12 mutations/Mb)) by using the lower bound value that covered the 90% probability interval across all MSI-H CRCs, and identified 2.9% of 5702 MSS cases were classified as TMB-H [26]. Here, we followed the definition of TMB-H as 10 mutations/Mb approved by the FDA and identified 53 MSS CRCs (~9%) with elevated TMB (13.6–153.8, Fig. 2b). This subgroup may also be beneficial from immune checkpoint inhibition. Besides the enrichment of *BRAF*^V600E^ mutation within MSI-high subgroup, other off-label markers in PI3Ki, PARPi, MEKi, and mTORi drug groups provides alterative targeted therapy choices for patients who fail ICI therapy (Fig. 4b). Furthermore, recent studies have shown other non-mutational influences may also dictate response to ICI in CRC including the presence of tumour-infiltration lymphocytes (TIL), certain tumour microenvironment (TME) gene expression patterns, and immunophenotypes [27, 28].

Taken together, our study evaluating the prioritisation of targeted therapies options for patients with recurrent late-stage CRC showed that about 51% of patients will be eligible to standard care actionability and about 49% of patients could be enrolled in clinical trials with investigational drugs. Furthermore, among patients with actionable alterations in RAS/RAF pathway, one-third of the right-sided tumours, and one-fifth of the left-sided tumours could benefit from combined therapies that simultaneously target RAS/RAF and PI3K pathways. In addition, there are several limitations of this study: (1) Actionable gene fusions were not assessed in this study cohort. (2) Gene methylation, promotor, and intronic regions cannot be assayed in this NGS platform, (3) Due to the low resolution of amplicon-based targeted NGS and the uneven distribution of amplicon number across the gene panel, CNV detection may be ambiguous. Therefore, the clinical utility of amplicon-based targeted NGS for unequivocal copy-number calculation and actionability assessment remains questionable. (4) The concordance between the NGS-based MSI algorithm and dMMR IHC method needs further evaluation. (5) The gene expression data for determining the consensus molecular subtypes, CMS1(immune), CMS2(canonical), CMS3(metabolic), and CMS4(mesenchymal) were not available.

## Supplementary information

check list

## Data Availability

The datasets used and/or analysed during this study are available from the corresponding author on reasonable request.
